# Evaluation of the paw withdrawal latency for the comparison between tramadol and butorphanol administered locally, in the plantar surface of rat, preliminary study

**DOI:** 10.1371/journal.pone.0254497

**Published:** 2021-07-26

**Authors:** Claudia Interlandi, Fabio Leonardi, Filippo Spadola, Giovanna Lucrezia Costa

**Affiliations:** 1 Department of Veterinary Sciences, University of Messina, Messina, Italy; 2 Department of Medicine and Veterinary Sciences, University of Parma, Parma, Italy; University of Bari, ITALY

## Abstract

The aim of the study was to evaluate the analgesic efficacy of tramadol compared to butorphanol administered locally in ventral surface of the hind paw of rats. Prospective, randomized experimental study; twenty-one adult male *Wistar rats* were selected. Heart rate (beats minute^-1^), respiratory rate (breaths minute^-1^), and paw withdrawal latency (onset of radiant heat until paw withdrawal/seconds) were measured prior (T^0^) and after (T^5^, T^10^, T^15^, T^20^) intraplantar injection with saline solution 0,9% (group S), butorphanol 1 mg kg^-1^ (group B), and tramadol 1 mg kg^-1^ (group T). Shapiro-Wilk normality test and Friedman test were used to analyze the data expressed by median and range. Statistical significance was set at p < 0.05. Statistical analysis of heart rate showed that there were significant differences between groups at different monitoring times. There were no significant differences in respiratory rate after intraplantar injection in any of the treatment groups. The paw withdrawal latency values at T5, T10, and T15 minutes after intraplantar injection in the group B were significantly higher compared to baseline value and to the values of the other groups. The paw withdrawal latency were no significant changes in the measurements of intragroup in S and T. Intraplantar administration of butorphanol provides a good analgesia and significantly increases paw withdrawal latency compared to tramadol. Intraplantar injection of butorphanol could be useful and safe and safe technique to achieve local analgesia for minor surgical procedures in rats.

## Introduction

Local analgesia is considered a valuable aid to general anaesthesia during various surgical procedures.

Specific research is needed to study the local effects of analgesic agents, that usually are used systemically [1, 2].

Opioids were used as adjuvants with local anesthetics to hasten the onset action and prolong duration and it is also known that intrathecal opioids administration produce a dose-dependent visceral analgesia [3–5].

For this purpose, two well-known analgesic drugs (i.e. tramadol and butorphanol), have been used and compared. Butorphanol is commonly used to sedate dogs, cats and horses, and is a κ-opioid receptor agonist and a μ-opioid receptor antagonist [6, 7]. It activates (G)-protein-coupled receptors at the level of the central nervous system by a reduction of cyclic adenosine monophosphate, which leads to the suppression of sodium and calcium channels. This mechanism of action leads to membrane hyperpolarization and transmission suppression of the pain ascending pathways.

Tramadol is an atypical opioid consisting of two enantiomers that inhibit the re-uptake of serotonin and noradrenaline. It modulates the inhibitory descending pathways and has a good analgesic efficacy combined with minor side effects. Furthermore, tramadol produces only mild sedative effects and it is inexpensive [8].

Tramadol is usually administered to treat postoperative pain in humans, dogs and cats [9, 10]. Nevertheless, it has been recently demonstrated that tramadol also provides analgesia in farm animals [11]. Moreover, tramadol is also currently considered for analgesic management in more animal species as well as humans [11–14].

In fact, intraplantar injections of tramadol have previously shown encouraging results for use in local anaesthesia for Wistar rats [5, 15].

The aims of this study were to evaluate and compare the effects of these two analgesic drugs administered via intraplantar injections and to promote their use in the minor surgery of domestic animals.

## Materials and methods

The study was carried at the University of Messina out in 2009 and was approved by the Review Board for Animals Care of the Department of Health and Human Services of Italy, with the formula "silence / consent". Animal care was in accordance with Italian regulations on the protection of animals used for experimental and other scientific purposes (D.M.116192), as well as with EEC regulations (O.J. of E.C. L 358/1 12/18/1986).

This study was performed on Wistar male rats (400–500 g, 7 weeks old) supplied by Envigo RMS Srl, S. Pietro al Natisone, Udine, Italy.

Rats were housed in individual polycarbonate cages and maintained under a 12:12 light–dark cycle at 21 ± 1 °C with 50 ± 5% humidity. The cage bedding material was corn cob and was changed twice per week. Food and water were available ad libitum.

All experiments were performed between 9:00 a.m. and 4:00 p.m., husbandry conditions and overall welfare-related animal assessments were respected.

The rats were acclimatised in the polycarbonate cages before the experiment.”

After the experiment, the rats were placed in polycarbonate cages and anaesthetised with ketamine 50 mg kg^-1^ (Ketavet®10% MSD Animal Health S.r.l.) in association with dexmedetomidine 0.05 mg kg^-1^ (Dexdomitor® 0.5mg/ml Zoetis MX) given by intramuscular injection. They were then suppressed with Tanax® (Intervet srl, Italy) administered by intracardiac injection at 2 ml/patient.

Death was confirmed after cessation of vital signs. Euthanasia was unrelated to the study.

The rats evaluated for the study totalled 30. Of these, nine subjects were excluded for the following reasons: foot or other part of the body injured and being underweight. Therefore, 21 rats were included in the project.

Rats (n = 21) were divided into three groups, control group (n = 7 –group S) to which a saline solution 9% (S.A.L.F. spa Laboratorio Farmacologico, Italy) was administered and the other two groups were injected respectively (n = 7 –group T) with tramadol 1mg kg^-1^ (Altadol, Formenti, Italy) and (n = 7 –group B) with butorphanol 1mg kg^-1^ (Dolorex, Intervet, Italy).

Data recorded, included heart rate (HR), respiratory rate (*f*_R_) and paw withdrawal latency (PWL), was collected before drug administration (basal values, T^0^), and at 5(T^5^), 10(T^10^), 15(T^15^) and 20 minutes (T^20^) after intraplantar injection. The rats included were randomly assigned and blind data recording were performed.

During the experimental phase, the paw withdrawal latency (PWL) was recorded. Rats were placed in a transparent plexiglass container with a clear smooth glass floor (Ugo Basile Thermal Plantar, Rats & Mice Italy). A radiant heat source mounted on a movable holder below the clear smooth glass floor was placed directly under the plantar surface of the left or right hind paws.

Heart rates (HR) were measured using 8 MHz vascular Doppler (UltraTec PD1v, Ultrasound technologies, UK), positioned on the left heart area, and respiratory rates (*f*_R_) were obtained by the direct observation of thoracic wall excursions.

Local analgesia was evaluated through a thermal-nociceptive sensitivity to radiant heat (40°C) using the PWL [1, 5, 16–18]. The PWL was defined as the time in seconds from exposure onset radiant heat until paw withdrawal.

In addition, evaluation of analgesia was performed with a cumulative pain scale assigning a score on percentage variations of heart rate (HR, beats minute^-1^), and respiratory rate (*f*_R,_ breaths minute^-1^), compared with basal values, as following:

0 ≤ 0%
1 = > 0% but ≤ 10%
2 = > 10% but ≤ 20%
3 = > 20% but ≤ 30%
4 = > 30%

The total score is obtained from the sum of the percentage variations of HR and *f*_R,_ According Numeric Rating scale a total score greater than 10 was indicative of severe pain [7, 19]. Group S (n = 7) received 0.9% sterile saline solution (SALF, Italy), group B (n = 7) received butorphanol 1 mg kg^-1^ (Dolorex, Intervet, Italy) and group T (n = 7) received tramadol 1 mg kg^-1^ (Altadol, Formenti, Italy).

The drugs used were diluted in normal saline (pH = 5.5) at a same volume of 0.05 mL/rat. For drug administration rats were placed in DecapiCones (tapered plastic film tubes) and injected with a 27 G needle under the ventral surface of the right hind paw.

Statistical analyses were performed using software package SPSS 15.0 (IBM Company, Italy). Data were expressed by median and range. Shapiro-test normality testing was performed.

Changes globally were evaluated with a Friedman test. Statistical significance was set at *p* < 0.05.

## Results

The paw withdrawal latency (PWL) showed significant differences in the three groups at all time of the study (*p* = 0.001).

Friedmann testing showed no significant differences at T^0^ between groups B, S and T.

The differences were highly significant between the groups; B and S (*p* = 0.006), T and S (*p* = 0.005) and B and T (*p* = 0.001).

Statistical differences were found in intragroup B (*p* = 0.015).

However, there were no significant changes in the measurements of the S and T on an intragroup basis [Table 1].

**Table 1:** 

Variable	Treatment	Time points (minutes)				
		T0	T5	T10	T15	T20
HR (beats/min^.-1^)	S	210 (168/222) *	168 (138/258) *	192 (174/204) *	180 (162/192) *	216 (186/240) *
	B	198 (156/222) *	192 (150/234) *	210 (168/234) *	222 (186/240) *^♣^^▽^	228 (192/240) *^▽^
	T	264 (246/330) *	240 (222/276) * ^ϯ^ ° ^▽^	282 (252/360) * ^ϯ^ °	270 (252/324) * ^ϯ^ °	270 (216/300) * ^ϯ^ °
*f*r (breaths/min^.-1^)	S	124 (96/144)	92 (72/134)	112 (76/124)	96 (80/124)	100 (92/128)
	B	104 (88/104)	110 (104/112)	108 (88/124)	104 (92/132)	100 (88/120)
	T	108 (56/152)	104 (56/144)	116 (84/124)	100 (88/136)	116 (88/156)
PWL (plantar withdrawal latency/seconds)	S	14 (11/20)	16 (5/20) *	15 (7/23) *	16 (5/25) *	13 (5/20)
	B	10,6 (3/17)	32,3 (6/32) * ^♣^^▽^	25,4 (13/32) * ^♣^^▽^	32,3 (9/32) * ^♣^^▽^	10,3 (6/25)
	T	12,8 (5/14)	7,8 (3/12) * °	7,6 (6/12) * °	11,6 (6/15) * °	7,2 (6/15)

The experiments were performed between 9:00 a.m. and 4:00 p.m.;

Median and Range, of physiological parameters, in Wistar rats, after administration of saline solution 9% (group S), butorphanol 1mg kg^-1^ (group B), and tramadol 1mg kg^-1^ (group T). HR—heart rate; *f*_R_—respiratory rate; PWL—paw withdrawal latency;

*Statistically different between groups (*p* < 0.05);

^♣^ Statistically different between group S and group B (*p* < 0.05);

^ϯ^ Statistically different between group S and group T (*p* < 0.05);

° Statistically different between group B and group T (*p* < 0.05);

^▽^Statistically different in intragroup (*p* < 0.05).

We observed that the rats belonging to group T, after analgesic drug injection, were excited and continuously moving inside their cages.

With regard to heart rate (HR), the differences between the groups globally were very significant (*p* = 0,001), and the difference was already present at the basal measurement (*p* = 0,004).

Differences were significant, at all times point monitored, between group S and group B (p = 0.001), group S and group T (p = 0.001) and the groups B and T (p = 0.001) [Table 1].

The Friedman test in group T showed a significant variations over the timeline (*p* = 0.039). In particular, we founded significant difference at T^5^ (*p* = 0.049)

Along the timeline there were also significant variations in group B (*p* = 0.039),

where HR were significantly increased at T^15^ (*p* = 0,049) and T^20^ (*p* = 0.046).

Unlike the other groups, along the timeline of group S, there were no significant difference [Table 1].

The respiratory rate (*f*_R_) did not show any difference between the groups globally (*p* = 0.173).

According to the findings obtained in the current survey no statistically significant differences in cumulative pain score based on percentage variations of HR and *f*_R_ were found [Table 2].

**Table 2:** 

Group	Time points (minutes)			
	T^5^	T^10^	T^15^	T^20^
S	0 (0/3)	0 (0/3)	0 (0/2)	0 (0/4)
B	1 (0/4)	1 (0/4)	1 (0/4)	0 (0/2)
T	0 (0/3)	1,5 (0/4)	0 (0/4)	1 (0/4)

Evaluation of analgesia was performed with a cumulative pain scale (CPS) assigning a score on percentage variations of heart rate (HR), and respiratory rate (*f*_R_), compared with basal values; the total score is obtained from the sum of the percentage variations of HR and *f*_R_, total score greater than 10 was indicative of severe pain; the results obtained evidenced that there is no significant difference between all groups in the times monitored”

## Discussion

In this study, the plantar test has been used to assess the hind-paw nociceptive withdrawal latencies to thermal stimuli in rat [16].

The various ways of analgesic drug administration must be adapted to the different needs of the patients, depending on animal species, age, health status, type of surgery and degree of pain [7].

Associating local anaesthesia with an analgesic drug allows it to have a good local effect by reducing any central side effects that butorphanol and tramadol (injected generally) could cause [5]. This is important since the use of long-acting anaesthetics can trigger an inflammatory reaction and increase postsurgical pain [1].

Our results showed that butorphanol, at a dose of 1 mg kg^-1^, produced good local analgesia associated with a stable clinical condition at all the monitored time points. After an intraplantar injection of butorphanol, the heart and respiratory rates remained within the physiological ranges at all the monitored time points. Whereas, the paw withdrawal latency time was significantly increased when compared to the other groups. During monitoring, the rats moved slowly and reacted less to their surroundings, which suggests that butorphanol could have had both local and systemic effects, providing also a mild sedation.

Butorphanol has been previously used by local administration with encouraging results [4, 20].

The heart rates of the group treated with tramadol had higher basal values at T^0^ when compared to the other groups.

Respiratory rate did not show any significant changes during monitoring.

Paw withdrawal latency values showed significant differences between groups T and B and between groups B and S, while there were no differences between groups T and S.

Surprisingly, in subjects treated with tramadol, we highlighted a state of agitation that was associated with a greater responsiveness of the subject. On the contrary, these behavioural changes were not noticed in rats belonging to the other groups.

It will be necessary to investigate whether this state of agitation is caused by a local irritating action [8, 18, 21] or a systemic effect of the drug [22].

In other species (bovine and horse), we previously noticed that a single dose of tramadol quickly administered caused a paradoxical effect. The effect was not recorded when tramadol was administered at the same dose by continuous infusion [13, 22].

In fact, from our results, it is not clear whether there was any analgesic effect from tramadol. This finding is in contrast to previous studies that reported a good degree of local analgesia, with higher doses of tramadol [1] and others who reported lower doses [5], compared with the dose used in the present study. Furthermore, it could be important to establish an optimal dose of tramadol to promote local analgesia [5, 12, 18].

Based on various studies, it could be supposed that the effect of tramadol is related to the dose, route of administration (intravenous or intraplantar) and method of administration (fast or slow bolus). Therefore, all of these factors may affect the degree of antinociception.

In human beings, the use of tramadol in local anaesthesia is very encouraging, with good results being obtained, for example, for pain management in children undergoing tonsillectomy and in submucous local tramadol in the surgical removal of mandibular third molars [9, 10, 23, 24].

In mice it was reported that a lower analgesic effect in tramadol compared to lidocaine, after the local administration (ventral surface of hindpaw) of glutamate, induced allodynia. This case would be a decreased efficacy of tramadol relative to lidocaine on damaged peripheral nerves [18]. However, it has also been reported that tramadol alone, locally administered in mice, produced its analgesic effects in the tail-flick test by immersion of the tail in hot water, which is indicative of a local anaesthetic action by blockade of the sodium channels [18, 25].

Intraplantar administration of tramadol alone, or combined with codeine, in perineural anaesthesia also produced good local analgesia in rats [1, 5, 15].

Further studies are needed to assess the analgesic efficacy of tramadol administered via intraplantar injection.

Based on our experience, the use of butorphanol at a dose of 1 mg kg^**-1**^ for local analgesia produces good analgesia for about 15 minutes in rats.

## Conclusion

In conclusion, based on the results obtained in this study we can conclude that the use of butorphanol may be useful and safe strategy to ensure a good local analgesia The action of tramadol is still controversial, and further studies regarding the mode of administration are needed.

It will be interesting to continue investigations to enrich analgesic protocols and to extend its use to other animal species.

## Supporting information

S1 Data. Minimal data regarding the effects of tested drugs (i.e. tramadol, T; butorphanol, B) on paw withdrawal latency, heart and respiratory rate recorded from twenty-one adult male Wistar rats (XLS)
